# Food Insecurity Is Associated with Mild Cognitive Impairment among Middle-Aged and Older Adults in South Africa: Findings from a Nationally Representative Survey

**DOI:** 10.3390/nu11040749

**Published:** 2019-03-30

**Authors:** Ai Koyanagi, Nicola Veronese, Brendon Stubbs, Davy Vancampfort, Andrew Stickley, Hans Oh, Jae Il Shin, Sarah Jackson, Lee Smith, Elvira Lara

**Affiliations:** 1 Research and Development Unit, Parc Sanitari Sant Joan de Déu, Universitat de Barcelona, Fundació Sant Joan de Déu, 08830 Barcelona, Spain; 2 Instituto de Salud Carlos III, Centro de Investigación Biomédica en Red de Salud Mental, CIBERSAM, 28029 Madrid, Spain; elvira.lara@uam.es; 3 ICREA, Pg. Lluis Companys 23, 08010 Barcelona, Spain; 4 Aging Branch, Neuroscience Institute, National Research Council, 35128 Padova, Italy; ilmannato@gmail.com; 5 National Institute of Gastroenterology “S. De Bellis” Research Hospital, Castellana Grotte, 70013 Bari, Italy; 6 Physiotherapy Department, South London and Maudsley NHS Foundation Trust, Denmark Hill, London SE5 8AZ, UK; brendon.stubbs@kcl.ac.uk; 7 Department of Psychological Medicine, Institute of Psychiatry, Psychology and Neuroscience, King’s College London, London WC2R 2LS, UK; 8 Faculty of Health, Social Care and Education, Anglia Ruskin University, Chelmsford CM1 1SQ, UK; 9 Department of Rehabilitation Sciences, KU Leuven, 3001 Leuven, Belgium; davy.vancampfort@kuleuven.be; 10 University Psychiatric Center KU Leuven, KU Leuven, 3000 Leuven, Belgium; 11 Department of Preventive Intervention for Psychiatric Disorders, National Institute of Mental Health, National Center of Neurology and Psychiatry, Kodaira, Tokyo 187-8553, Japan; amstick66@gmail.com; 12 The Stockholm Center for Health and Social Change (SCOHOST), Södertörn University, 141 89 Huddinge, Sweden; 13 School of Social Work, University of Southern California, CA 90015, USA; hansoh@usc.edu; 14 Department of Pediatrics, Yonsei University College of Medicine, Yonsei-ro 50, Seodaemun-gu, C.P.O. Box 8044, Seoul 03722, Korea; SHINJI@yuhs.ac; 15 Division of Pediatric Nephrology, Severance Children’s Hospital, Seoul 03722, Korea; 16 Institute of Kidney Disease Research, Yonsei University College of Medicine, Seoul 03722, Korea; 17 Department of Behavioural Science and Health, University College London, London WC1E 7HB, UK; s.e.jackson@ucl.ac.uk; 18 The Cambridge Centre for Sport and Exercise Sciences, Anglia Ruskin University, Cambridge CB1 1PT, UK; Lee.Smith@anglia.ac.uk; 19 Department of Psychiatry, Hospital Universitario de La Princesa, Instituto de Investigación Sanitaria Princesa (IIS-Princesa), 28006 Madrid, Spain

**Keywords:** mild cognitive impairment, food insecurity, South Africa, epidemiology

## Abstract

There are no studies on the association between food insecurity and mild cognitive impairment (MCI). Thus, cross-sectional, community-based data on individuals aged ≥50 years from the World Health Organization’s Study on Global AGEing and Adult Health (SAGE) conducted in South Africa (2007–2008) were analyzed to assess this association. The definition of MCI was based on the National Institute on Ageing-Alzheimer’s Association criteria. Past 12-month food insecurity was assessed with two questions on frequency of eating less and hunger due to lack of food. Multivariable logistic regression analysis was conducted. The sample consisted of 3,672 individuals aged ≥50 years [mean (SD) age 61.4 (18.3); 56% females]. The prevalence of MCI was 8.5%, while 11.0% and 20.8% experienced moderate and severe food insecurity, respectively. After adjustment for potential confounders, moderate and severe food insecurity were associated with 2.82 (95%CI = 1.65–4.84) and 2.51 (95%CI = 1.63–3.87) times higher odds for MCI compared with no food insecurity, respectively. The OR for those aged ≥65 years with severe food insecurity was particularly high (OR = 3.87; 95%CI = 2.20–6.81). In conclusion, food insecurity was strongly associated with MCI among South African older adults. Future longitudinal research is required to assess whether addressing food insecurity may reduce risk of MCI and subsequent dementia.

## 1. Introduction

Dementia is a debilitating syndrome that results in the deterioration of memory, thinking, behavior, and the ability to conduct daily activities. It is one of the main causes of disability and dependency among the older adult population globally [1]. The 2016 Global Burden of Disease (GBD) study showed that 28.8 million disability-adjusted life years (DALYs) are attributed to dementia, and that dementia is the fifth leading cause of death globally [2]. Worldwide, it has been estimated that approximately 50 million people have dementia, of which about 60% live in low- and middle-income countries (LMICs), while there are nearly 10 million new cases each year [3]. As a consequence of global aging, the total number of people with dementia is expected to triple from its current figure by 2050 and reach 152 million, with this increase being largely attributable to rising numbers in LMICs [3]. Despite the overwhelming burden of dementia, especially in the years to come, there are no truly disease-modifying treatments for dementia [4]. Thus, there is a crucial need to identify modifiable risk factors for the preclinical transitional stages of dementia such as mild cognitive impairment (MCI). MCI has a high progression rate to dementia (12%, 20%, and 50% at 1, 3, and 5 years, respectively [5]), and is increasingly being considered as an important stage for intervention to prevent or delay the onset of dementia.

Currently, there is increasing evidence that food insecurity is associated with cognitive decline [6,7,8]. Food insecurity is defined as “limited or uncertain availability of nutritionally adequate and safe foods or limited or uncertain ability to acquire food in socially acceptable ways” [9]. It has been hypothesized that food insecurity may increase risk of cognitive decline via stress, depression, or poor nutritional intake [8]. However, there are no studies specifically on the association between food insecurity and MCI, or studies on food insecurity and any form of cognitive impairment from LMICs despite the fact that food insecurity is more common in this setting [10].

South Africa is an apposite setting in which to examine the association between food insecurity and MCI as the level of food insecurity has been reported to be high in this country [11], which may be attributable to the extremely high levels of absolute poverty compared with other middle-income countries [12]. Furthermore, rapid urbanization, the HIV epidemic, and increasing food prices are also likely to be implicated in the high rate of food insecurity in South Africa [11,12,13]. Finally, an extremely high prevalence of obesity and hypertension have been reported in South Africa [14], and these factors can potentially contribute to an upward trend in dementia in the future as these conditions are known to be risk factors for dementia [15]. Thus, the aim of the current study was to assess whether food insecurity is associated with MCI using data from a nationally representative sample of older adults in South Africa collected as part of the Study on Global AGEing and Adult Health (SAGE). 

## 2. Materials and Methods 

Data from the SAGE survey conducted in South Africa between 2007–2008 were analyzed. This dataset is publically available to all interested researchers via the WHO website (http://www.who.int/healthinfo/sage/en/) upon request. Detailed sampling information can be found in the above-mentioned WHO website. Briefly, a stratified multistage cluster sampling design was used to obtain a nationally representative sample. Strata were defined by the nine provinces (Eastern Cape, Free State, Gauteng, Kwa-Zulu Natal, Limpopo, Mpumalanga, North West, Northern Cape and Western Cape), locality (urban or rural), and predominant racial group (African/Black, White, Colored and Indian/Asian). Enumeration areas (EAs) constituted the primary sampling units (PSUs) and were selected with probability proportional to size: the measure of size being the number of individuals aged 50 or over in the EA. A different questionnaire was administered to a proxy respondent if the selected individual could not participate in the survey due to limited cognitive function. Information from proxies was not used in the current study. The survey response rate was 75%. Household weights were post-stratified by province and locality according to the South African Community Survey 2007. Individual weights were post-stratified by province, sex and age groups according to the 2009 Medium Mid-Year population estimates from Statistics South Africa. Ethical approval was obtained from the WHO Ethical Review Committee and Human Sciences Research Council, Pretoria, South Africa. All participants provided written informed consent.

### 2.1. Mild Cognitive Impairment (MCI)

We used the recommendations of the National Institute on Aging-Alzheimer’s Association to define MCI [16]. The exact same algorithms used in past SAGE publications were applied [17,18]. Briefly, MCI was defined as fulfilling all of the following conditions: 

(a) Concern regarding cognitive changes: This condition referred to replying ‘bad’ or ‘very bad’ to the question “How would you best describe your memory at present?” and/or answering ‘worse’ to the question “Compared to 12 months ago, would you say your memory is now better, the same or worse than it was then?”

(b) Objective evidence of impairment in at least one cognitive domain: The following performance tests were used to assess cognitive function: word list immediate and delayed verbal recall based on the Consortium to Establish a Registry for Alzheimer’s Disease [19], which evaluated learning and episodic memory; digit span forward and backwards based on the Weschler Adult Intelligence Scale [20], that assessed working and attention memory; and the animal naming task [19], which evaluated verbal fluency. Individuals with a level of performance that was below -1 SD after adjustment for education and age on at least one of these tests were considered to have this condition.

(c) Preserved independence in functional abilities: Questions on self-reported past-30-day difficulties with basic activities of daily living (ADL) were used to assess this condition [21]. Specifically, the questions were: “How much difficulty did you have in getting dressed?” and “How much difficulty did you have with eating (including cutting up your food)?” The response options included none, mild, moderate, severe, and extreme (cannot do). Independence in functional activities was considered to be preserved if the participant answered either none, mild, or moderate to both of these questions. All other participants were omitted from the analysis (83 individuals aged ≥50 years).

(d) Absence of dementia: Individuals who could not participate in the survey due to severe cognitive impairment were excluded from the current study.

### 2.2. Food Insecurity

Food insecurity was defined with the use of the two following questions: “In the last 12 months, how often did you ever eat less than you felt you should because there wasn’t enough food?” and “In the last 12 months, were you ever hungry, but didn’t eat because you couldn’t afford enough food?” Both of these questions had as response options: every month (coded = 1); almost every month (coded = 2); some months, but not every month (coded = 3); only in 1 or 2 months (coded = 4); never (coded = 5). These items were based on similar items found in food security questionnaires such as the US Household Food Security Survey Module and National Health and Nutrition Examination Survey (NHANES) Food Security module. As in a previous SAGE study, those who answered 1 through 3 to both questions or answered 1 to either item were categorized as severely food insecure. Those who did not fulfill the criteria for severe food insecurity but answered 2 through 4 for either question were coded as moderately food insecure. Those who answered 5 to both items were categorized as food secure [22].

### 2.3. Control Variables

Past literature was used as a guide to select the control variables [7]. Specifically, these included sex, age (years), years of education, wealth quintiles based on income, race (Black, White, other), low physical activity, smoking (never, past, current), alcohol use in the past 30 days, past 12 month DSM-IV depression, body mass index (BMI) based on measured weight and height [<18.5 (underweight), 18.5–24.9 (normal), 25.0–29.9 (overweight), ≥30 kg/m^2^ (obese)], and chronic physical conditions (diabetes, hypertension, stroke). Stroke and diabetes were based only on lifetime self-reported diagnosis. Hypertension referred to having self-reported diagnosis, systolic blood pressure ≥140 mmHg, or diastolic blood pressure ≥90 mmHg. The Global Physical Activity Questionnaire was used to assess the level of physical activity [23]. Low physical activity was defined as <150 minutes of moderate-to-vigorous physical activity in a typical week [24]. The endorsement of DSM-IV depression was based on the World Mental Health Survey version of the Composite International Diagnostic Interview [25].

### 2.4. Statistical Analysis

The statistical analysis was done with Stata 14.1 (Stata Corp LP, College station, TX, USA). The analysis was limited to those aged ≥50 years. Middle-aged individuals were also included in the current study because cognitive dysfunction can appear up to 10 years prior to dementia diagnosis [26], and intervening in mid-life is now considered crucial [27]. The difference in sample characteristics by level of food insecurity was tested by Chi-squared tests and Student’s *t*-tests for categorical and continuous variables, respectively.

We conducted multivariable logistic regression analysis to assess the association between food insecurity (exposure) and MCI (outcome) in the overall sample (i.e., age ≥50 years) and by age group (50–64 and ≥65 years) as the risk factors for MCI may differ between mid-life and late-life [28]. The regression analysis was adjusted for age, sex, education, wealth, race, physical activity, smoking, alcohol use, BMI, diabetes, stroke, hypertension, and depression. Given that some authors have suggested that depression may be an important mediator in the association between food insecurity and cognitive decline [8], we also constructed a model without adjustment for depression using the overall sample to assess the degree to which the association between food insecurity and MCI is explained by depression. All variables were included in the regression analysis as categorical variables with the exception of age and years of education (continuous variables). The sample weighting and the complex study design were taken into account in the analyses. Results from the regression analyses are presented as odds ratios (ORs) with 95% confidence intervals (CIs). The level of statistical significance was set at *p* < 0.05. 

## 3. Results

The final analytical sample comprised 3,672 individuals aged ≥50 years [mean (SD) age 61.4 (18.3) years; 56% females]. The proportion of those aged 50–64 and ≥65 years was 66.8% and 33.2%, respectively. Overall, the prevalence of MCI was 8.5% (95%CI = 6.9–10.3), while the prevalence of moderate (unweighted *n* = 332) and severe (unweighted *n* = 677) food insecurity was 11.0% (95%CI = 9.0–13.4) and 20.8% (95%CI = 17.9–23.9), respectively. The sample characteristics are shown in Table 1. Compared to respondents without food insecurity, those who were food insecure were significantly more likely to have less education and wealth, and lower levels of physical activity, and be of Black race. 

**Figure 1:**
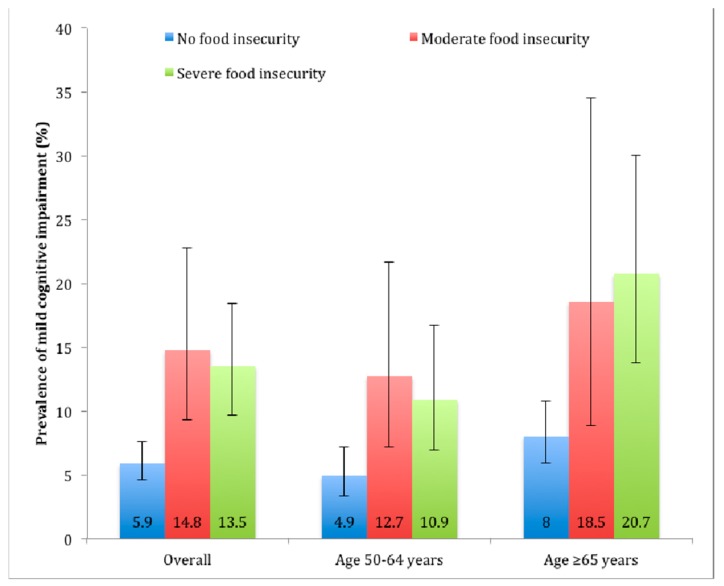
Prevalence of mild cognitive impairment by food insecurity status. Estimates are based on weighted sample. Bars denote 95% confidence interval.

The prevalence of MCI was higher among those with food insecurity than in those without food insecurity (Figure 1). For example, overall, the prevalence of MCI among those without food insecurity was 5.9% but this increased to 14.8% and 13.5% among those with moderate and severe food insecurity, respectively.

The association between food insecurity and MCI estimated by multivariable logistic regression is shown in Table 2. After adjustment for a variety of sociodemographic and behavioral factors as well as physical health conditions and depression, compared to no food insecurity, moderate and severe food insecurity were associated with 2.82 (95%CI = 1.65–4.84) and 2.51 (95%CI = 1.63–3.87) times higher odds for MCI, respectively, in the overall sample. These estimates were not substantially different from the model that did not include depression with the corresponding figures being 2.83 (95%CI = 1.65–4.85) and 2.58 (95%CI = 1.68–3.97), respectively (data shown only in text). The OR among those aged ≥65 years with severe food insecurity was particularly elevated (OR = 3.87; 95%CI = 2.20–6.81).

## 4. Discussion

In our study on community-dwelling adults aged ≥50 years in South Africa, we found a high prevalence of food insecurity (31.8%). After adjustment for potential confounders, moderate and severe food insecurity were associated with significant 2.82 and 2.51 times higher odds for MCI, while the OR was particularly elevated for severe food insecurity among those aged ≥65 years (OR = 3.87). Depression was not a major explanatory factor in this association. 

To the best of our knowledge, this is the first study to specifically examine the relationship between food insecurity and MCI. Our findings are in line with previous studies that have examined the association between food insecurity and cognitive function in the USA although these studies were on general cognitive function and not MCI. One community-based cross-sectional study conducted among a representative sample of 1358 Puerto Ricans aged 45–75 years living in Massachusetts found that the adjusted difference in the Mini-Mental State Examination (MMSE) score was 0.90 lower among those with very low food security as compared to those who were food secure [8]. A longitudinal follow-up study among 597 participants aged 40–75 years based on the same study population found that food insecurity at baseline was associated with lower global cognitive function after two years of follow-up [7]. Finally, one cross-sectional US study using data from the NHANES found that among 1851 adults between 60 and 85 years, food insecurity was associated with poor cognitive function [6]. 

The exact mechanisms linking food insecurity and MCI are unknown, but there are several hypotheses. It is possible that stress resulting from food insecurity may increase the risk of MCI. For example, prolonged elevation of cortisol (an HPA axis response to chronic stress) can lead to alterations in brain structure and function (e.g., in the hippocampus) and subsequent cognitive decline [29]. Also, increases in pro-inflammatory cytokines induced by stress [30] may lead to an increased risk of dementia [31]. Furthermore, food insecurity often compromises diet quality, as people tend to switch to more affordable but less nutritious food when food is scarce (e.g., high fat and carbohydrates, low vitamins and micronutrients) [32]. Poor diet has been associated with increased risk of cognitive decline. For example, vitamin B, C, and E have all been shown to have a protective effect against dementia [33]. Furthermore, randomized clinical trials have shown that supplementation of n-3 polyunsaturated fatty acids [34] and folic acid [35] may improve cognitive function in older people with MCI. Finally, high carbohydrate intake has been associated with higher risk of MCI [36]. 

Our finding that a particularly strong association was observed among older individuals concurs with that of the study by Gao and colleagues [8], which found that the difference in MMSE scores between very low food insecurity and food secure were −1.75 and −0.48 for those aged ≥60 and <60 years, respectively [8]. This age difference may be related to increased vulnerability of the brain among the older population. For example, older adults have smaller hippocampal volumes and this may increase susceptibility to cognitive deficits when exposed to stress [37]. The fact that people aged ≥65 years had 3.87 times higher odds for MCI compared with those without food insecurity is an important finding as the risk of dementia is particularly elevated in this age group [38].

Strengths of the study include the large sample size and the use of nationally representative data. However, some limitations should be taken into consideration when interpreting the findings. First, participants with mild forms of dementia could have been included in our study sample as the study did not include a clinical assessment of dementia. Second, there is no consensus regarding the acceptable level of functional impairment in MCI [39]. The definition of preservation of independence in functional abilities used in our study, which has been used in previous publications [17,18,28,40], was rather conservative. This was done to avoid the omission of MCI cases with disability not related to their cognitive ability. Despite these potential limitations, it is worth noting that the prevalence of MCI in our study was consistent with previously reported figures [41]. Third, data on biomarkers such as cerebrospinal fluid Aβ and tau were not available. These data could have provided a better understanding on how food insecurity affects brain pathology [4]. Fourth, the estimates in the analyses stratified by age should be interpreted with caution as the sample size was small and some estimates had wide confidence intervals. Next, we lacked information on HIV infection, which has been reported to be associated with a higher risk of food insecurity [42] and impaired cognitive function [43]. However, given that the link between food insecurity and HIV infection is likely to be mainly explained by poverty [42], we believe that the adjustment for wealth in our study is likely to have minimized the potential for residual confounding due to HIV infection. Furthermore, our measure of food insecurity was based on two questions and did not constitute a comprehensive food insecurity measure. Finally, because this was a cross-sectional study, causality cannot be inferred. For example, it is possible that people with cognitive impairments have difficulty in utilizing social safety net services, and this might have led to food insecurity.

## 5. Conclusions

In conclusion, we found that the prevalence of food insecurity is high in South African older adults, and that food insecurity is associated with MCI, with this association being particularly pronounced among individuals aged ≥65 years. Although biological plausibility suggests that food insecurity may lead to MCI, future studies with a longitudinal and experimental design are warranted to assess causality and the utility of addressing food insecurity as a preventive strategy for MCI and subsequent dementia.

## Figures and Tables

**Table 1:** Sample characteristics (overall and by food insecurity).

Characteristic		Overall	Food Insecurity			*p*-Value ^a^
			None	Moderate	Severe	
Sex	Female	56.0	54.4	56.5	62.8	0.047
	Male	44.0	43.5	37.2	43.6	
Age (years)	Mean (SD)	61.4 (18.3)	61.7 (18.9)	62.5 (17.4)	60.1 (17.1)	0.074
Education (years)	Mean (SD)	6.1 (10.1)	6.8 (11.0)	4.5 (8.1)	4.7 (7.3)	<0.001
Wealth	Poorest	20.7	14.7	27.7	32.7	<0.001
	Poorer	19.9	17.9	20.8	26.4	
	Middle	18.5	17.7	25.1	19.8	
	Richer	19.8	22.1	20.3	13.2	
	Richest	21.1	27.6	6.0	7.9	
Race	Black	74.3	68.2	80.1	88.9	<0.001
	White	9.2	12.9	2.0	1.5	
	Other	16.5	19.0	17.9	9.6	
Low physical activity	No	50.0	54.1	39.5	41.9	0.002
	Yes	50.0	45.9	60.5	58.1	
Smoking	Never	66.6	66.1	69.0	66.7	0.162
	Past	23.8	23.3	20.2	27.3	
	Current	9.7	10.7	10.8	6.0	
Alcohol consumption	No	86.0	86.8	91.1	80.7	0.007
	Yes	14.0	13.2	8.9	19.3	
BMI (kg/m^2^)	Normal	24.0	23.3	21.5	25.8	0.382
	Overweight	26.5	27.8	22.1	25.5	
	Obese	46.6	46.4	51.9	45.1	
	Underweight	2.9	2.5	4.5	3.6	
Diabetes	No	90.9	91.3	88.1	90.5	0.465
	Yes	9.1	8.7	11.9	9.5	
Stroke	No	96.6	96.6	96.7	96.3	0.951
	Yes	3.4	3.4	3.3	3.7	
Hypertension	No	21.4	21.8	16.7	20.5	0.278
	Yes	78.6	78.2	83.3	79.5	
Depression	No	97.1	97.7	97.6	95.0	0.064
	Yes	2.9	2.3	2.4	5.0	

Abbreviation: SD Standard deviation; BMI Body mass index; Data are percentage unless otherwise stated. ^a^ *p*-value was calculated by Chi-squared tests and Student’s *t*-tests for categorical and continuous variables, respectively.

**Table 2:** Association of food insecurity and other covariates with mild cognitive impairment (outcome) estimated by multivariable logistic regression.

Characteristic	Category	Age					
		Overall		50–64 years		≥65 years	
		OR	95%CI	OR	95%CI	OR	95%CI
Food insecurity	None	1.00		1.00		1.00	
	Moderate	2.82 ***	[1.65,4.84]	2.84 **	[1.41,5.69]	2.76 *	[1.19,6.41]
	Severe	2.51 ***	[1.63,3.87]	1.98 *	[1.06,3.72]	3.87 ***	[2.20,6.81]
Sex	Female	1.00		1.00		1.00	
	Male	0.56 **	[0.37,0.85]	0.59	[0.32,1.06]	0.54	[0.26,1.10]
Age (years)		1.05 ***	[1.03,1.07]	1.05	[0.99,1.11]	1.04 *	[1.01,1.08]
Education (years)		0.98	[0.93,1.03]	0.95	[0.89,1.02]	1.01	[0.94,1.09]
Wealth	Poorest	1.00		1.00		1.00	
	Poorer	1.08	[0.64,1.83]	0.68	[0.34,1.36]	2.32	[0.99,5.40]
	Middle	1.51	[0.82,2.80]	1.06	[0.52,2.16]	2.95	[0.98,8.87]
	Richer	1.82	[0.96,3.46]	1.54	[0.73,3.27]	2.45	[0.87,6.93]
	Richest	1.35	[0.60,3.04]	1.12	[0.37,3.42]	2.11	[0.63,7.09]
Race	Black	1.00		1.00		1.00	
	White	0.23 *	[0.07,0.74]	0.18	[0.03,1.02]	0.24 *	[0.06,0.96]
	Other	0.87	[0.50,1.50]	0.92	[0.46,1.80]	0.58	[0.25,1.34]
Low physical activity	No	1.00		1.00		1.00	
	Yes	0.79	[0.52,1.20]	0.72	[0.40,1.31]	0.75	[0.45,1.23]
Smoking	Never	1.00		1.00		1.00	
	Past	1.11	[0.67,1.82]	1.45	[0.76,2.77]	0.71	[0.36,1.37]
	Current	1.15	[0.63,2.11]	1.16	[0.54,2.48]	1.1	[0.41,2.93]
Alcohol consumption	No	1.00		1.00		1.00	
	Yes	2.44 **	[1.39,4.28]	2.90 **	[1.49,5.64]	1.57	[0.71,3.48]
BMI (kg/m^2^)	Normal	1.00		1.00		1.00	
	Overweight	0.95	[0.53,1.69]	1.69	[0.82,3.49]	0.40 *	[0.18,0.89]
	Obese	1.17	[0.74,1.85]	1.63	[0.85,3.11]	0.61	[0.31,1.21]
	Underweight	0.89	[0.40,2.01]	0.92	[0.30,2.84]	0.76	[0.20,2.80]
Diabetes	No	1.00		1.00		1.00	
	Yes	0.9	[0.46,1.76]	0.97	[0.39,2.43]	0.87	[0.33,2.29]
Stroke	No	1.00		1.00		1.00	
	Yes	4.64 ***	[1.91,11.29]	5.02 **	[1.81,13.91]	2.92 *	[1.02,8.39]
Hypertension	No	1.00		1.00		1.00	
	Yes	0.9	[0.55,1.46]	1.17	[0.58,2.34]	0.56	[0.30,1.03]
Depression	No	1.00		1.00		1.00	
	Yes	1.77	[0.68,4.58]	2.41	[0.92,6.31]	0.58	[0.06,6.00]

Abbreviation: OR Odds ratio; CI Confidence interval; BMI Body mass index. Models are mutually adjusted for all variables in the Table. * *p* < 0.05, ** *p* < 0.01, *** *p* < 0.001.
